# The Association between High Mobility Group Box 1 and Stroke-Associated Pneumonia in Acute Ischemic Stroke Patients

**DOI:** 10.3390/brainsci12111580

**Published:** 2022-11-19

**Authors:** Yan E, Qiwen Deng, Guomei Shi, Zhongyuan Li, Chengfang Liu, Siyu Wang, Huiwen Lian, Haiming Cao, Xiaohao Zhang, Yingdong Zhang, Pengyu Gong, Teng Jiang

**Affiliations:** 1 Department of Neurology, Nanjing First Hospital, Nanjing Medical University, Nanjing 210006, China; 2 Department of Neurology, Taixing People’s Hospital, Taixing 225400, China; 3 Department of Neurology, The Affiliated Hospital of Nantong University, Nantong 226000, China

**Keywords:** acute ischemic stroke, high-mobility-group box 1, biomarker, stroke-associated pneumonia, restricted cubic spline

## Abstract

Objective: This study aimed to investigate the association between high-mobility-group box 1 (HMGB1) and stroke-associated pneumonia (SAP) in acute ischemic stroke (AIS) patients. Methods: AIS patients were enrolled in two centers. The serum samples were collected within the first 24 h after admission, and HMGB1 levels were measured by enzyme-linked immunosorbent assay. Logistic regression models were used to calculate the odds ratio (OR) and 95% confidence interval (95% CI) of SAP for HMGB1 concentrations. Restricted cubic splines (RCS) were performed to explore the shapes of the association between HMGB1 concentrations and SAP. Results: From January 2022 to May 2022, a total of 420 AIS patients were enrolled. Ninety-six (22.9%) patients develop SAP. The levels of HMGB1 in the SAP group were higher than those in the non-SAP group (*p* < 0.001). Using the first quartile of HMGB1 group as a reference, patients in the fourth quartile of HMGB1 group had the highest likelihood of experiencing SAP in the unadjusted model (OR = 3.687; 95% CI: 1.851–7.344), age- and sex-adjusted model (OR = 3.511; 95% CI: 1.725–7.147), and multivariable-adjusted model (OR = 2.701; 95% CI: 1.045–6.981). HMGB1 was also independently associated with SAP as a continuous variable in the unadjusted model (OR = 1.132; 95% CI: 1.069–1.199), age- and sex-adjusted model (OR = 1.131; 95% CI: 1.066–1.200), and multivariable-adjusted model (OR = 1.096; 95% CI: 1.011–1.188). RCS showed a linear association between HMGB1 and SAP (*p* for linear trend = 0.008) Conclusions: HMGB1 might be able to act as a potential biomarker of SAP in AIS patients.

## 1. Introduction

In China, ischemic stroke serves as one of the main reasons for mortality and long-term disability in elderly people [1,2,3]. However, approximately 7–38% of acute ischemic stroke (AIS) patients suffer from stroke-associated pneumonia (SAP), which may lead to unfavorable prognosis and disability [4,5,6,7,8,9]. Hence, it is valuable and meaningful to explore novel biomarkers for SAP prediction in AIS patients.

In recent years, the studies have highlighted the role of inflammation in brain disorders [10,11,12,13,14,15]. As a DNA-binding protein, high-mobility-group box 1 (HMGB1) is expressed in all cell types [16]. Based on the previous findings, extracellular HMGB1 acts as a damage-associated molecular-pattern molecule and promotes inflammatory response [16,17,18]. So far, HMGB1 has been considered a useful biomarker for severity stratification and prognosis prediction in various diseases, such as acute myocardial infarction and idiopathic pulmonary fibrosis [19,20]. More recently, Shan et al. revealed that higher HMGB1 levels in the acute phase of ischemic stroke were associated with increased risk of post-stroke depression [21]. To date, few clinical studies have focused on the relationship between HMGB1 concentrations and SAP. Accordingly, this study was conducted to investigate whether HMGB1 could serve as a biomarker for SAP in AIS patients.

## 2. Materials and Methods

### 2.1. Study Design and Participants

AIS patients were enrolled from Nanjing First Hospital and Taixing People’s Hospital. All the AIS patients were treated in the stroke units and received standard treatments. Eligible participants were recruited in the final analysis if they met the following criteria. Informed consent was obtained from participants or their legal representatives. This study was approved by the Ethics Committee of Nanjing First Hospital and Taixing People’s Hospital.

Inclusion criteria:

(1) Admission within 48 h after onset of AIS,
(2) Age 18 years or older.

Exclusion criteria:

(1) Pneumonia or active infection before admission;
(2) Dysphagia before admission;
(3) Preventive antibiotic therapy;
(4) Received endovascular treatment;
(5) Hospitalization lasts less than 7 days;
(6) Incomplete clinical data.

### 2.2. Data Acquisition

On the day of admission, all the participants underwent standard assessments of demographic characteristics (age, sex, and lower literacy level (primary school or lower)), vascular risk factors (hypertension, diabetes mellitus, atrial fibrillation, coronary heart disease, and previous stroke), clinical characteristics (stroke severity, dysphagia, blood pressure, body mass index (BMI), pulse, and intravenous thrombolysis), stroke subtype, and laboratory data. The severity of stroke was assessed by the National Institutes of Health Stroke Scale (NIHSS) score. Systolic blood pressure (SBP) and diastolic blood pressure (DBP) were measured and recorded shortly after admission. Computed tomography (CT), magnetic resonance (MR), electrocardiogram, echocardiography, carotid ultrasonography, and transcranial doppler were performed to assess stroke subtype. Stroke subtype was classified according to Trial of Org 10172 in Acute Stroke Treatment (TOAST) criteria [22]. Laboratory data included white blood cells (WBC), fasting blood glucose (FBG), total cholesterol (TC), triglycerides (TG), high-density lipoprotein (HDL), low-density lipoprotein (LDL), and uric acid.

### 2.3. Diagnosis of SAP

The diagnosis of SAP was based on the modified Centers for Disease Control and Prevention criteria of hospital-acquired pneumonia by two trained clinicians, according to clinical and laboratory parameters of acute lower respiratory tract infection, and was confirmed by chest X-ray or CT [6,23,24].

### 2.4. Detection of HMGB1 Concentrations

Blood samples were collected within the first 24 h after admission. Serum HMGB1 concentrations were measured with a commercially available enzyme-linked immunosorbent assay kit (30164033, Hycult Biotech, Uden, the Netherlands). Laboratory technicians who performed these measurements were blind to the clinical characteristics and outcomes of the study participants.

### 2.5. Statistical Analysis

Statistical analyses were performed with SPSS version 25.0 (SPSS Inc.) and R version 4.2.1 software which accessed on 24 August 2022 (http://www.R-project.org/). Categorical variables were expressed as n (%). Continuous variables were expressed as means (standard deviation, SD) or medians (interquartile range, IQR). Differences in baseline characteristics between groups were analyzed using t-tests or Mann–Whitney U tests for continuous variables as well as the Chi-squared test, likelihood ratio test, or Fisher’s exact test for categorical variables, as appropriate. The violin plot was used to display the distribution of HMGB1 concentrations between the SAP group and the non-SAP group. Three logistic regression models were used to calculate the odds ratio (OR) and 95% confidence interval (95% CI) of SAP for the higher quartile of HMGB1 concentrations compared to the lowest quartile and for HMGB1 concentrations as continuous variables. Model 1 was the unadjusted model. Model 2 was adjusted for age and sex. Model 3 was adjusted for age, sex, and other variables with *p* < 0.1 in the univariate analysis. Restricted cubic splines (RCS) were performed to explore the shapes of the associations between HMGB1 concentrations and SAP with three knots (at the 5th, 50th, and 95th percentiles). A two-tailed value of *p* < 0.05 was considered significant.

## 3. Results

**Figure 1:**
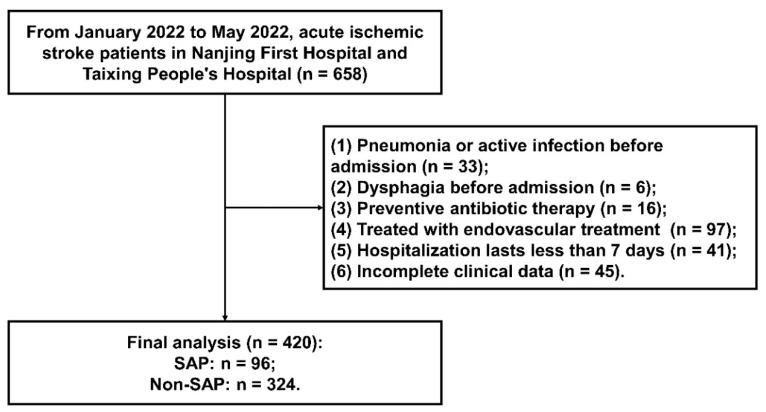
The flowchart of participant selection.

The flowchart of participant selection from January 2022 to May 2022 is described in Figure 1. A total of 658 AIS patients were screened. Eventually, a total of 420 AIS participants were enrolled in this observational research. Patients were excluded due to the following reasons: pneumonia or active infection before admission (n = 33), dysphagia before admission (n = 6), preventive antibiotic therapy (n = 16), endovascular treatment (n = 97), hospitalization lasting less than 7 days (n = 41), and incomplete clinical data (n = 45). Ultimately, there were 96 (22.9%) AIS patients that developed SAP. The baseline characteristics of the SAP group and the non-SAP group are shown in Table 1. Significant differences between the two groups are described as follows: age (*p* < 0.001), atrial fibrillation (*p* < 0.001), coronary heart disease (*p* = 0.046), previous stroke (*p* = 0.012), NIHSS at admission (*p* < 0.001), dysphagia at admission (*p* < 0.001), ventilator during hospitalization (*p* < 0.001), BMI (*p* = 0.045), TOAST subtype (*p* = 0.013), WBC (*p* < 0.001), and FBG (*p* < 0.001).

**Figure 2:**
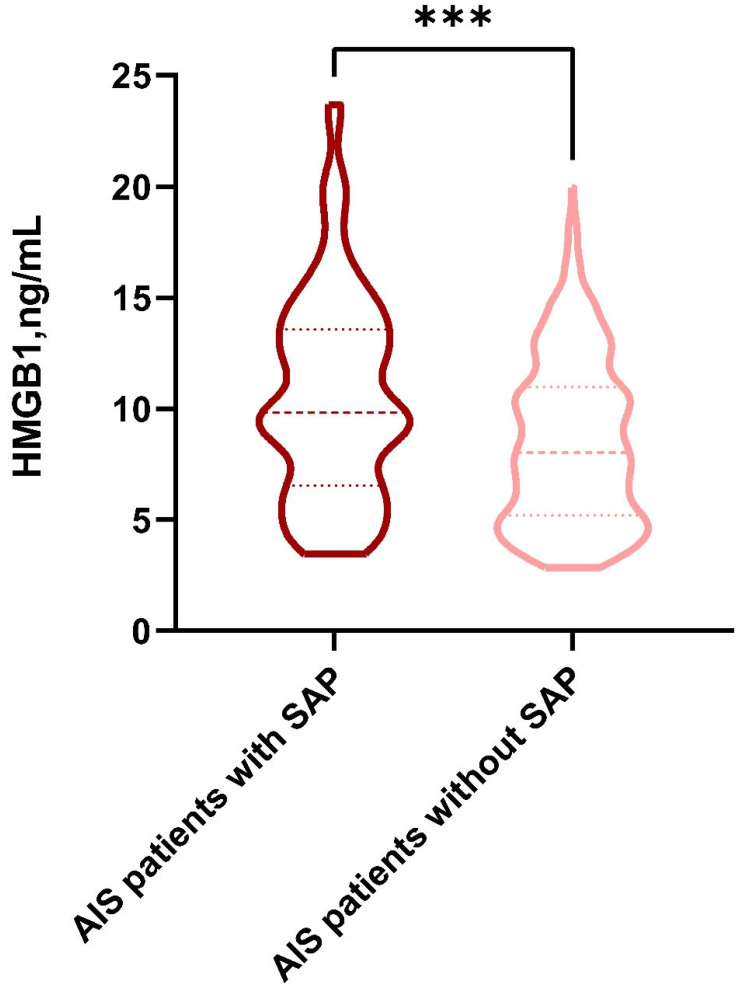
The violin plot in the distribution of HMGB1 concentrations in the SAP group and the non-SAP group. HMGB1, high-mobility-group box 1; SAP, stroke-associated pneumonia. ***: *p* < 0.001.

The distribution of HMGB1 concentrations in the SAP group and the non-SAP group is indicated by Figure 2. The levels of HMGB1 in the SAP group were higher than those in the non-SAP group (9.83 [6.54, 13.57] versus 8.03 [5.19, 10.98], respectively; *p* < 0.001). 

**Figure 3:**
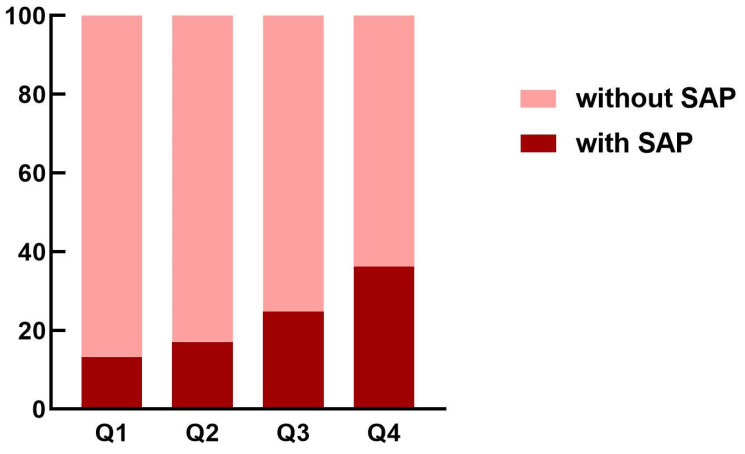
The proportion of SAP in acute ischemic stroke patients according to the quartiles of HMGB1 concentrations. HMGB1, high-mobility-group box 1; SAP, stroke-associated pneumonia.

As shown in Figure 3, the incidence of SAP was elevated among patients in the higher quartile of the HMGB1 group than those in the lower quartile of the HMGB1 group. As indicated by Table 2, patients in the fourth quartile of the HMGB1 group had the highest likelihood of experiencing SAP (OR = 3.687; 95% CI: 1.851–7.344; unadjusted model, using the first quartile of HMGB1 group as reference). This association remained significant in the age- and sex-adjusted model (OR = 3.511; 95% CI: 1.725–7.147) as well as the multivariable-adjusted model (OR = 2.701; 95% CI: 1.045–6.981). HMGB1 was also independently associated with SAP as a continuous variable in the unadjusted model (OR = 1.132; 95% CI: 1.069–1.199), age- and sex-adjusted model (OR = 1.131; 95% CI: 1.066-1.200), and multivariable-adjusted model (OR = 1.096; 95% CI: 1.011–1.188).

**Figure 4:**
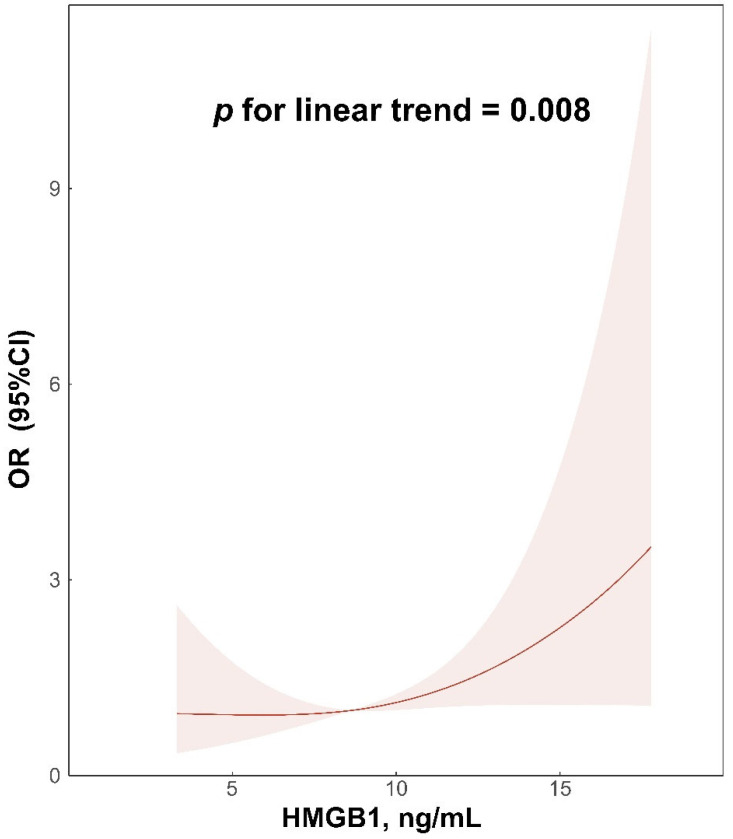
Relationship of HMGB1 concentrations with SAP in acute ischemic stroke patients. Odds ratios and 95% confidence intervals derived from restricted cubic spline regression, which was adjusted for age, sex, atrial fibrillation, coronary artery disease, previous stroke, NIHSS at admission, dysphagia at admission, ventilator during hospitalization, BMI, TOAST subtype, WBC, and FBG, with knots placed at the 5th, 50th, and 95th percentiles of the distribution of HMGB1 concentrations. HMGB1, high-mobility-group box 1; SAP, stroke-associated pneumonia; NIHSS, National Institute of Health Stroke Scale; BMI, body mass index; TOAST, Trial of Org 10172 in Acute Stroke Treatment; WBC, white blood cells; FBG, fasting blood glucose.

In the multivariable-adjusted RCS regression, HMGB1 concentrations exhibited a linear association with SAP (*p* for non-linear trend = 0.358, *p* for linear trend = 0.008; Figure 4) in AIS patients.

## 4. Discussion

In this study, we found that HMGB1 levels at admission, either as categorical or continuous variables, were associated with SAP in AIS patients. In addition, multivariable-adjusted RCS regression showed the linear association between the baseline levels of HMGB1 and SAP. Our findings indicated that HMGB1 might be considered a biomarker of SAP in AIS patients.

According to previous studies, HMGB1 is a nuclear protein that exists in almost all kinds of nucleated animal cells [25]. HMGB1 can be passively released by necrotic or damaged cells from the nucleus to the extracellular space. After ischemic insults, fully reduced HMGB1 translate into disulfide HMGB1 [26,27] and elicit neuroinflammation via toll-like receptors, receptor for advanced glycation end products, or other receptors [28]. HMGB1 can also interact with matrix metalloproteinase enzymes and thus lead to the disruption of the blood–brain barrier [26,29]. These specific effects of HMGB1 contribute to the pathogenesis of cerebral ischemic injury.

Although the clinical value of HMGB1 has been confirmed in numerous diseases, studies focusing on the association of HMGB1 concentrations with SAP in patients with ischemic stroke have rarely been carried out. According to a previous study by Huang et al., the levels of HMGB1 were markedly elevated in patients with type 2 diabetes mellitus combined with chronic obstructive pulmonary disease [30]. In Italian diabetes mellitus patients, HMGB1 levels were related with an increased risk of carotid-plaque vulnerability, which may result in stroke occurrence [31]. Meanwhile, Wang et al. found that HMGB1 levels might be linked to functional outcomes and mortality in AIS patients treated with intravenous thrombolysis [32]. In addition, a meta-analysis showed that ischemic stroke patients possessed elevated levels of HMGB1 compared with healthy controls, and the levels of HMGB1 were positively associated with severity and infarct volume in ischemic stroke patients [33]. Moreover, a recent study showed that higher baseline levels of HMGB1 were related to increased risk of post-stroke depression [21]. In the current study, we showed that patients with elevated levels of HMGB1 at admission were more likely to develop SAP. This association remained significant after adjusting for potential confounding factors. What is more, there was a linear association between HMGB1 concentrations and SAP in AIS patients according to the results of RCS. To our knowledge, this is the first study revealing that HMGB1 might be a biomarker of SAP in AIS patients.

It should be noted that this study has some limitations. First, the sample size of this study is relatively small, and we plan to carry out prospective studies with larger samples to further validate these findings. Second, we only collected samples within 24 h of stroke onset to detect the levels of HMGB1. Dynamic examination of HMGB1 is warranted to be performed in our future research.

In conclusion, this study provides the first evidence that HMGB1 is able to act as a potential biomarker of SAP in AIS patients. These findings may help clinicians in risk stratification of SAP in AIS patients. In future, prospective studies with larger samples are warranted to further validate this conclusion.

## Figures and Tables

**Table 1:** Characteristics of all patients referred with SAP.

Characteristics	Non-SAP (n = 324)	SAP (n = 96)	*p*
Demographics			
Age, years	65.7 ± 11.9	72.8 ± 12.2	<0.001
Male, n (%)	222 (68.5)	64 (66.7)	0.732
Lower literacy level (primary school or lower), n (%)	107 (33.0)	35 (36.5)	0.532
Vascular risk factors, n (%)			
Hypertension	217 (67.0)	68 (70.8)	0.477
Diabetes mellitus	98 (30.2)	33 (34.4)	0.443
Atrial fibrillation	33 (10.2)	25 (26.0)	<0.001
Coronary heart disease	33 (10.2)	17 (17.7)	0.046
Previous stroke	54 (16.7)	27 (28.1)	0.012
Clinical characteristics			
NIHSS at admission	3 (2, 4)	9 (5, 16)	<0.001
Dysphagia at admission, n (%)	21 (6.5)	51 (53.1)	<0.001
SBP, mmHg	143.7 ± 19.7	144.5 ± 24.0	0.720
DBP, mmHg	85.9 ± 12.6	85.4 ± 13.1	0.769
Ventilator during hospitalization, n (%)	1 (0.3)	13 (13.5)	<0.001
BMI, kg/m^2^	24.8 ± 3.3	23.9 ± 4.1	0.045
Pulse, /min	78 (70, 80)	78 (69, 89)	0.223
Intravenous thrombolysis, n (%)	78 (24.1)	31 (32.3)	0.107
TOAST subtype, n (%)			0.013
LAA	146 (45.1)	42 (43.8)	
CE	43 (13.3)	27 (28.1)	
SAO	113 (34.9)	22 (22.9)	
SOE	12 (3.7)	3 (3.1)	
SUE	10 (3.1)	2 (2.1)	
Laboratory data			
WBC, 10^^9^/L	7.29 (5.95, 8.93)	9.92 (7.04, 12.91)	<0.001
FBG, mmol/L	5.42 (4.77, 6.63)	6.25 (5.22, 7.80)	<0.001
TC, mmol/L	4.43 ± 1.14	4.41 ± 1.17	0.834
TG, mmol/L	1.34 (0.98,1.78)	1.25 (0.80, 1.70)	0.122
HDL, mmol/L	1.05 (0.90, 1.29)	1.16 (0.86, 1.49)	0.115
LDL, mmol/L	2.56 (1.93, 3.24)	2.49 (1.90, 3.16)	0.573
Uric acid, ng/mL	307.5 (252.0, 369.8)	314.5 (254.0, 397.8)	0.263

Abbreviations: SAP, stroke-associated pneumonia; NIHSS, National Institute of Health Stroke Scale; SBP, systolic blood pressure; DBP, diastolic blood pressure; BMI, body mass index; TOAST, Trial of Org 10172 in Acute Stroke Treatment; LAA, large-artery atherosclerosis; CE, cardioembolism; SAO, small-artery occlusion; SOE, stroke of other determined etiology; SUE, stroke of undetermined etiology; WBC, white blood cells; FBG, fasting blood glucose; TC, total cholesterol; TG, triglycerides; HDL, high-density lipoprotein; LDL, low-density lipoprotein.

**Table 2:** Odds ratio and 95% confidence interval of SAP according to HMGB1 among acute ischemic stroke patients.

	HMGB1 as a Categorical Variable				HMGB1 as a Continuous Variable
	Q1	Q2	Q3	Q4	
Case, n (%)	14 (13.3)	18 (17.1)	26 (24.8)	38 (36.2)	96 (22.9)
Unadjusted model	1.00 (reference)	1.345 (0.630–2.869)	2.139 (1.045–4.378)	3.687 (1.851–7.344)	1.132 (1.069–1.199)
Age- and sex-adjusted	1.00 (reference)	1.344 (0.618–2.924)	2.025 (0.967–4.241)	3.511 (1.725–7.147)	1.131 (1.066–1.200)
Multivariable-adjusted *	1.00 (reference)	1.139 (0.409–3.169)	1.019 (0.360–2.886)	2.701 (1.045–6.981)	1.096 (1.011–1.188)

* Adjusted for age, sex, and other variables with *p* < 0.1 in Table 1, including atrial fibrillation, coronary artery disease, previous stroke, NIHSS at admission, dysphagia at admission, ventilator during hospitalization, BMI, TOAST subtype, WBC, and FBG. Abbreviations: NIHSS, National Institute of Health Stroke Scale; BMI, body mass index; TOAST, Trial of Org 10172 in Acute Stroke Treatment; WBC, white blood cells; FBG, fasting blood glucose.

## Data Availability

The data that support the findings of this study are available from the corresponding author upon reasonable request.
